# Photocatalytic CO_2_ Reduction and Electrocatalytic H_2_ Evolution over Pt(0,II,IV)-Loaded Oxidized Ti Sheets

**DOI:** 10.3390/nano10101909

**Published:** 2020-09-24

**Authors:** Ju Hyun Yang, So Jeong Park, Choong Kyun Rhee, Youngku Sohn

**Affiliations:** 1 Department of Chemistry, Chungnam National University, Daejeon 34134, Korea; mil03076@naver.com (J.H.Y.); jsjs5921@naver.com (S.J.P.); ckrhee@cnu.ac.kr (C.K.R.); 2 Department of Chemical Engineering and Applied Chemistry, Chungnam National University, Daejeon 34134, Korea

**Keywords:** energy recycling, photocatalytic CO_2_ reduction, electrochemical hydrogen evolution, Ti oxide, Pt oxidation state

## Abstract

Energy recycling and production using abundant atmospheric CO_2_ and H_2_O have increasingly attracted attention for solving energy and environmental problems. Herein, Pt-loaded Ti sheets were prepared by sputter-deposition and Pt^4+^-reduction methods, and their catalytic activities on both photocatalytic CO_2_ reduction and electrochemical hydrogen evolution were fully demonstrated. The surface chemical states were completely examined by X-ray photoelectron spectroscopy before and after CO_2_ reduction. Gas chromatography confirmed that CO, CH_4_, and CH_3_OH were commonly produced as CO_2_ reduction products with total yields up to 87.3, 26.9, and 88.0 μmol/mol, respectively for 700 °C-annealed Ti under UVC irradiation for 13 h. Pt-loading commonly negated the CO_2_ reduction yields, but CH_4_ selectivity was increased. Electrochemical hydrogen evolution reaction (HER) activity showed the highest activity for sputter-deposited Pt on 400 °C-annealed Ti with a HER current density of 10.5 mA/cm^2^ at −0.5 V (vs. Ag/AgCl). The activities of CO_2_ reduction and HER were found to be significantly dependent on both the nature of Ti support and the oxidation states (0,II,IV) of overlayer Pt. The present result could provide valuable information for designing efficient Pt/Ti-based CO_2_ recycle photocatalysts and electrochemical hydrogen production catalysts.

## 1. Introduction

Recycle energy production using abundant CO_2_ and H_2_O has been a challenging project for energy and environment solutions. Hydrogen production via water splitting is another useful project for these types of solutions. For CO_2_ reduction into fuels, increasing selectivity and production efficiency have been very important issues [1,2,3,4,5]. Many strategies have been employed such as defect controlling [6], crystal facet tailoring [7,8], metal alloys/cocatalysts [9,10], support [11], and hybridization [12]. For hydrogen production, diverse strategies have also been employed [13,14,15,16]. Among various metal nanoparticles (NPs) on metal oxide supports such as TiO_2_, Pt NP has been the most studied and known to have very high beneficial catalytic activity to CO_2_ reduction as well as hydrogen production [17,18,19,20,21,22,23,24,25]. Fang et al. prepared Pt-loaded TiO_2_ spheres (>500 μm) by a modified TiO_2_ sol-gel method and microwave-assisted Pt reduction method using ethylene glycol as the reducing agent [19]. They reported CO_2_ reduction yields of CO and CH_4_ with 18 and 3.5 μmol g^−1^ h^−1^, respectively. Liu et al. prepared well-dispersed Pt on ultrathin TiO_2_ and showed 47 μmol of CH_4_ formation and 35 μmol of CO formation after UV light irradiation for 10 h [20]. Zhang et al. reported accumulated CH_4_ production yield to be about 2.0 μmol g^−1^ for Pt (0.12 *w*/*w*%)-loaded TiO_2_ catalyst at a reaction temperature of 342 K for 13 h [21]. Wang et al. prepared ultrafine Pt (0.5–1 nm)/TiO_2_ nanostructures on ITO via aerosol chemical vapor deposition for TiO_2_ and gas-phase sputtering deposition for Pt NPs [22]. They reported CO_2_ photoreduction efficiency of 1361 μmol g^−1^ h^−1^ for CH_4_ formation and 200 μmol g^−1^ h^−1^ for CO formation. Kometani et al. tested CO_2_ reduction over Pt-loaded TiO_2_ under supercritical condition of water and CO_2_ at 400 °C and 30 MPa [23]. They reported that CO (35.3 ppm), CH_4_ (42.7 ppm), and H_2_ (65.3 ppm) were major products, and HCOOH (1.7 μM) and HCHO (0.6 μM) were minor. Xiong et al. showed that CO_2_ reduction efficiency was highly dependent on the Pt-loading method, which determined the particle size, distribution, and Pt(0)/Pt(II) ratio [26]. They loaded Pt NPs on TiO_2_ with co-exposed {101} and {001} facets by photo-reduction and NaBH_4_-reduction methods using Pt(NH_3_)_4_Cl_2_ or H_2_PtCl_6_ as precursors. The highest (4.6 μmol g^−1^ h^−1^) and poorest (0.2 μmol g^−1^ h^−1^) CH_4_ yields were reported for Pt NPs by chemical reduction methods using Pt(NH_3_)_4_Cl_2_ and H_2_PtCl_6_, respectively. For crystal facet effects, Mao et al. reported that TiO_2_ with {010} facets showed a higher CO_2_ photoreduction performance (1.6 μmol g^−1^ h^−1^ of CH_4_) than that (0.8 μmol g^−1^ h^−1^) of TiO_2_ with {001} facets [27]. However, 1% Pt-loading on TiO_2_ with {001} facets showed a higher activity (2.6 μmol g^−1^ h^−1^) than that (1.2 μmol g^−1^ h^−1^) of Pt-loading on TiO_2_ with {010} facets. For catalyst and cocatalyst enhancements, Zhang et al. co-decorated Au and Pt NPs (5–12 nm) on TiO_2_ nanofibers (NFs) through an electrospinning method [10]. They reported (0.03 μmol h^−1^)/(0.06 μmol h^−1^), (0.31 μmol h^−1^)/(0.20 μmol h^−1^), (0.42 μmol h^−1^)/(0.08 μmol h^−1^), and (0.57 μmol h^−1^)/(0.09 μmol h^−1^) of CH_4_ production/CO production after UV-irradiation for 3 h for pure TiO_2_ NFs, Au/TiO_2_ NFs, Pt/TiO_2_ NFs, and Au_0.25_/Pt_0.75_/TiO_2_ NFs, respectively. The CH_4_ production was increased by 14 and 19 times after Pt and Au_0.25_/Pt_0.75_ loadings, respectively. The Pt-TiO_2_ based photocatalysts for CO_2_ reduction in the previously reported literatures were further discussed in comparison with the present result below.

Pt NPs on TiO_2_ has also been a good model system for photocatalytic and electrochemical hydrogen production [28,29,30,31,32,33]. Briefly, Lian developed Pt*^n^*^+^ (*n* = 0, 2, or 3)-Ti^3+^/TiO_2_ system and showed that the photocatalytic H_2_ evolution efficiency was higher than that of metallic Pt-loaded Ti^3+^/TiO_2_ and commercial P25 [33]. They attributed the higher efficiency to well dispersed Pt*^n^*^+^–O species, facilitating a photogenerated charge transfer. Yu et al. prepared Pt-loaded TiO_2_ and showed that both H_2_ and C_2_H_6_ products were produced by a photocatalytic reaction in a catalyst-dispersed water system filled with CH_4_ gas [29].

Herein, to further investigate the roles of overlayer Pt and Ti supports, we prepared sputter-deposited Pt (Pt-sp) and Pt NPs (using a reducing agent) over oxidized Ti sheets and tested for both photocatalytic CO_2_ reduction and electrochemical hydrogen production. Different oxidation states (0,II,IV) of Pt were obtained using two different Pt-loading methods. Different natures of Ti supports were obtained by using different thermal annealing treatments. Two different application tests were employed to examine both positive and negative roles in two different application areas. Thereby, the present results provide very useful information on design of catalysts for energy production via CO_2_ reduction and electrochemical hydrogen evolution.

## 2. Materials and Methods

### 2.1. Preparation of Sputter-Deposited Pt on Ti Sheets and Pt NP on Ti Sheets

Ti sheets (GR2, 32 mm × 32 mm, 0.1 mm thick) were cleaned by ultrasonication in acetone and isopropyl alcohol repeatedly, and dried under an IR lamp. Afterwards, ozone cleaning (UVC-150 ozone cleaner, Omniscience Co., Yongin, Kyoungki, Korea) was performed for 15 min. Ti sheets were then annealed at 400 or 700 °C for 2 h. After the thermal treatment, the sheets were also repeatedly cleaned by the same procedure above. Pt NPs were prepared by a reduction method using NaBH_4_ and sodium citrate solution for 24 h at room temperature. After that, the NPs were fully washed and drop-coated on the Ti substrate. The sample was abbreviated as Pt-NP/Ti. Pt deposition on the oxidized Ti sheet was performed by a sputter-coating method with an ionization current of 3 mA for 10 s using a SPT-20 ion sputter coater (COXEM Co., Daejeon, Korea). The calibrated Pt thicknesses was 0.5 nm, abbreviated as Pt-sp. The samples examined here were bare Ti, Ti (400 °C), Ti (700 °C), Pt-NP/Ti, Pt-NP/Ti (400 °C), Pt-NP/Ti (700 °C), Pt-sp/Ti, Pt-sp/Ti (400 °C), and Pt-sp/Ti (700 °C).

### 2.2. Characterisation of the Samples

The surface morphology of the sheets before and after CO_2_ reduction was examined using a Hitachi S-4800 scanning electron microscope (SEM) (Hitach Ltd., Tokyo, Japan) at an accelerating voltage of 10 kV and a current of 10 mA. The surface chemical states for oxidized Ti sheet and Pt-loaded Ti sheets before and after CO_2_ reduction were examined by taking X-ray photoelectron spectra (survey, Pt 4f, Ti 2p, C 1s, and O 1s) using a using a Thermo-VG Scientific K-alpha^+^ spectrometer (Thermo VG Scientific, Waltham, MA, USA) with a monochromatic Al *K*_α_ X-ray source and a hemispherical energy analyzer.

### 2.3. Photocatalytic CO_2_ Reduction and Electrochemical Hydrogen Evolution

The photocatalytic CO_2_ reduction was performed in a stainless-steel reactor (volume ~40 mL) with a quartz window (3 mm thick and 45 mm diameter) on top. An oxidized Ti sheet or a Pt-loaded sheet was placed in the reactor with 40 μL (or 5 μL) deionized water beside the sheet. Afterwards, the reactor was fully flushed under a stream of pure (99.999%) CO_2_ gas for at least 5 min. After the reactor was filled with CO_2_ gas and the inlet and outlet valves were closed. For CO_2_ reduction experiment, the reactor was placed under four 15 W UVC (200–280 nm) lamps (a power density of 5.94 mW/cm^2^) for 13 h. Blank tests under dark condition were also performed to examine the precise role of light. The CO_2_ reduction gas products such as CO, CH_4_, and CH_3_OH were analyzed by a YL 6500 gas chromatograph (Young In Chromass Co., Ltd., Seoul, Korea) equipped with a Ni catalyst methanizer assembly, a flame ionization detector (FID), and a thermal conductivity detector (TCD). For GC analysis, 0.5 mL volume of gas from the reactor was taken using a gastight syringe to be injected into two different columns of 40/60 Carboxen-1000 (Sigma-Aldrich, St. Louis, MO, USA) and HP-PlotQ-PT (Agilent Technologies, Inc., Santa Clara, CA, USA). Electrochemical hydrogen evolution reaction (HER) tests were performed using a three-electrode system (a Pt counter electrode, a Ag/AgCl reference electrode, and a Ti electrode working electrode) using a WPG100 Potentiostat/Galvanostat (WonATech Co., Ltd., Seoul, Korea) electrochemical workstation. Cyclic voltammetry (CV) and linear sweep voltammetry (LSV) were carried in 0.1 M H_2_SO_4_ electrolyte solution at a potential range from +0.1 to −0.8 V.

## 3. Results and Discussion

**Figure 1:**
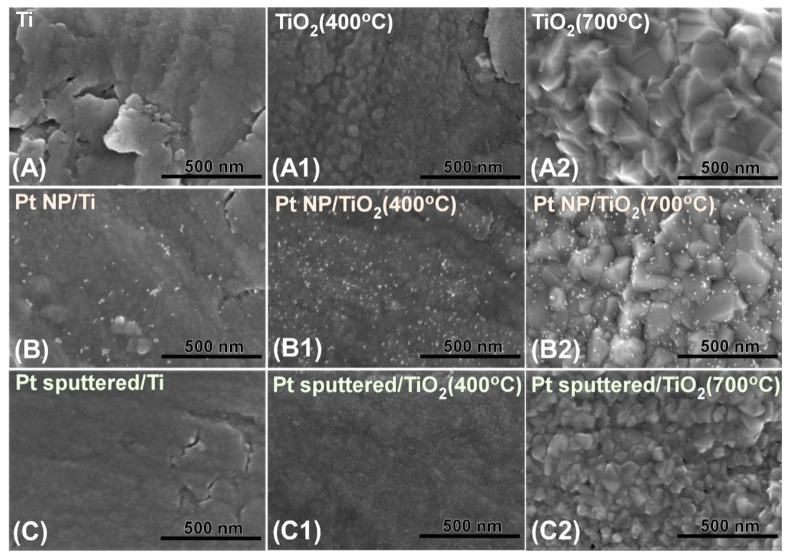
Scanning electron microscope (SEM) images of: (**A**) bare Ti; (**A1**) bare Ti (400 °C); (**A2**) bare Ti (700 °C); (**B**) Pt NP/Ti; (**B1**) Pt NP/Ti (400 °C); (**B2**) Pt NP/Ti (700 °C); (**C**) Pt-sp/Ti; (**C1**) Pt-sp/Ti (400 °C); (**C2**) Pt-sp/Ti (700 °C) samples.

Figure 1 shows the SEM images for bare Ti, oxidized (400 and 700 °C-annealed) Ti, Pt NP/Ti, Pt NP/Ti (400 °C), Pt NP/Ti (700 °C), Pt-sp/Ti, Pt-sp/Ti (400 °C), and Pt-sp/Ti (700 °C) samples. The surface morphology was found to be mainly determined by thermal treatment temperature and the Pt-prepared conditions. The samples (Figure 1(A2,B2,C2)) treated at 700 °C showed a more crystalline structure on the surface. However, the as-received Ti (Figure 1A) showed a blurred image surface. The 400 °C annealed sample (Figure 1(A1)) showed the morphology between the two. Upon Pt NP loading (prepared using a reducing agent) on the three different surfaces (bare Ti, 400 and 700 °C-annealed Ti), the surface morphology shown was quite distinct. The Pt NPs were aggregated on bare Ti (Figure 1B), while the NPs on 400 and 700 °C-annealed Ti (Figure 1(B1,B2)) sheets were relatively well dispersed. For the sputter-deposited Pt, no discernible particles were found. This could be due to that Pt (0.5 nm thick) was evenly embedded to form Pt-O species, which was confirmed by XPS below.

**Figure 2:**
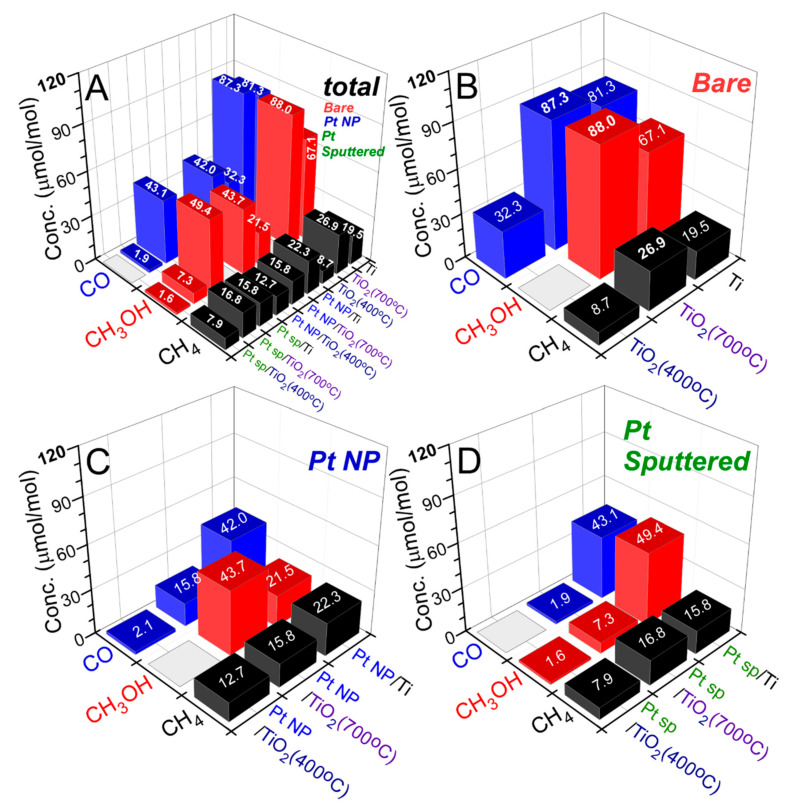
CO_2_ reduction (CO, CH_4_, and CH_3_OH) yields (μmol/mol) over: (**B**) bare Ti, Ti (400 °C), and Ti (700 °C); (**C**) Pt NP/Ti, Pt NP/Ti (400 °C), and Pt NP/Ti (700 °C); (**D**) Pt-sp/Ti, Pt-sp/Ti (400 °C), and Pt-sp/Ti (700 °C) samples. (**A**) is the total plot for (**B**–**D**).

For photocatalytic CO_2_ reduction, the design of a photoreactor system is also very important [1]. Two general photoreactor systems have been employed, which are (1) catalyst dispersed in a CO_2_-saturated aqueous liquid: solid-liquid mode, and (2) catalyst dispersed on a support with gaseous CO_2_ and H_2_O: solid-gas mode. In the present study, the solid (catalyst)-gas (CO_2_ + H_2_O) system was employed. CO_2_ reduction products were examined and displayed in Figure 2. Carbon monoxide (CO), methane (CH_4_), and methanol (CH_3_OH) were commonly been produced with different yields for bare Ti, Ti (400 °C), bare Ti (700 °C), Pt NP/Ti, Pt NP/Ti (400 °C), Pt NP/Ti (700 °C), Pt-sp/Ti, Pt-sp/Ti (400 °C), and Pt-sp/Ti (700 °C) sheets. For bare Ti, it was found that total CO, CH_3_OH, and CH_4_ production yields (μmol/mol = ppm) after UVC irradiation for 13 h were observed to be 81.3, 67.1, and 19.5 ppm, respectively. These yields became substantially decreased to 32.3, 0, and 8.7 ppm, respectively for Ti (400 °C) sheet. However, for 700 °C-annealed Ti, CO, CH_3_OH, and CH_4_ production yields became again increased to 87.3, 88.0, and 26.9 ppm, respectively. The CO/CH_4_ production ratios were estimated to be 4.2, 3.7, and 3.2 for bare Ti, Ti (400 °C), and Ti (700 °C), respectively. For bare Pt-free Ti substrates, the activity showed the order of Ti (400 °C) < Ti < bare Ti (700 °C). The Ti (700 °C) sheet showed the highest CO_2_ reduction activity. This indicates that the nature of Ti surface mainly determines the activity. Upon Pt-loading, the CO_2_ reduction yields were commonly diminished, but the CH_4_ production was less impacted. For Pt NP on Ti, the CO and CH_3_OH productions were decreased by 47% and 68%, respectively. However, CH_4_ production was increased by 14%. For sputter-deposited Pt on Ti, the CO, CH_3_OH, and CH_4_ productions were decreased by 48%, 26%, and 19%, respectively. For Pt NP on Ti (400 °C), CO and CH_4_ yields were observed to be 2.1 and 12.7 ppm, respectively compared with those of bare Ti (400 °C) sheet. CO yield was substantially decreased by 93%, but the CH_4_ yield was increased by 45%. For sputter-deposited Pt on Ti (400 °C), no CO was detected, and CH_3_OH and CH_4_ production yields were merely observed to be 1.6 and 7.9 ppm. For the Pt-loading on Ti (700 °C) sheet, the CO_2_ reduction activity was more severely impacted. CO, CH_3_OH, and CH_4_ production yields were decreased by 82%, 50%, and 41%, respectively. For the sputter-deposited Pt on Ti (700 °C), CO, CH_3_OH and CH_4_ production yields were more significantly decreased by 98%, 92%, and 38%, respectively. The CO/CH_4_ production ratios were estimated to be 0.16, 1.0, and 1.9 for Pt NP/Ti, Pt NP/Ti (400 °C), and Pt NP/Ti (700 °C), respectively. For Pt-sp/Ti, Pt-sp/Ti (400 °C), and Pt-sp/Ti (700 °C), the CO/CH_4_ production ratios were estimated to be 0.0, 0.1, and 2.7, respectively. CH_4_ selectivity was commonly increased upon Pt-loading.

CO_2_ reduction mechanism is highly dependent on the production of protons and electrons [1,10]. The general equation is written as *x*CO_2_ + *y*H^+^ + *z*e^−^ → C*_n_* products + *m*H_2_O, where C*_n_* is an organic compound. Under UV irradiation, electrons (e^−^) and holes (h^+^) are created in the conduction and valence bands (CB and VB), respectively. Because CO_2_ reduction occurs through multielectron processes, the creation of photogenerated electrons is an important factor for the catalytic activity. The photogenerated electrons and holes are separated to participate in several reactions described below:

TiO_2_ + hν → TiO_2_ (VB, h^+^) + TiO_2_ (CB, e^−^)

H_2_O + h^+^ → •OH + H^+^

•OH + H_2_O + 3h^+^ → O_2_ + 3H^+^

H^+^ + e^−^ → 1/2H_2_

CO_2_ + 2H^+^ + 2e^−^ → CO + H_2_O

CO + 6H^+^ + 6e^−^ → CH_4_ + H_2_O

CO_2_ + 6H^+^ + 6e^−^ → CH_3_OH + H_2_O

CO_2_ + 8H^+^ + 8e^−^ → CH_4_ + 2H_2_O

In the CO_2_ reduction mechanism, several reaction channels are closely spaced and diverse products of CO, CH_4_, and CH_3_OH are obtained via multielectron processes described above. Notably, kinetically favored hydrogen production channel is also among the CO_2_ reduction channels. Therefore, H^+^ and e^−^ can be consumed for H_2_ production. As discussed above, when Pt species were present, CO_2_ reduction was observed to be negated and photocatalytic hydrogen production was increased.

In Table 1, we have summarized Pt-TiO_2_ based photocatalysts for CO_2_ reduction in the previously reported literatures [10,17,19,20,21,22,24,27,34,35,36,37]. CO and CH_4_ have commonly been observed as CO_2_ reduction products, consistent with the present results. Generally, the yield of CH_4_ was reported to be higher than that of CO, although the reaction conditions (e.g., light intensity, wavelength, water amount, and reactor type) were all different. In the present result, CH_4_ production over Pt-loaded Ti oxides was also observed to be higher than CO production, which was consistent with the literature. This indicates that the CH_4_ production channel became superior to the CO production channel upon Pt-loading, which is discussed further below.

**Figure 3:**
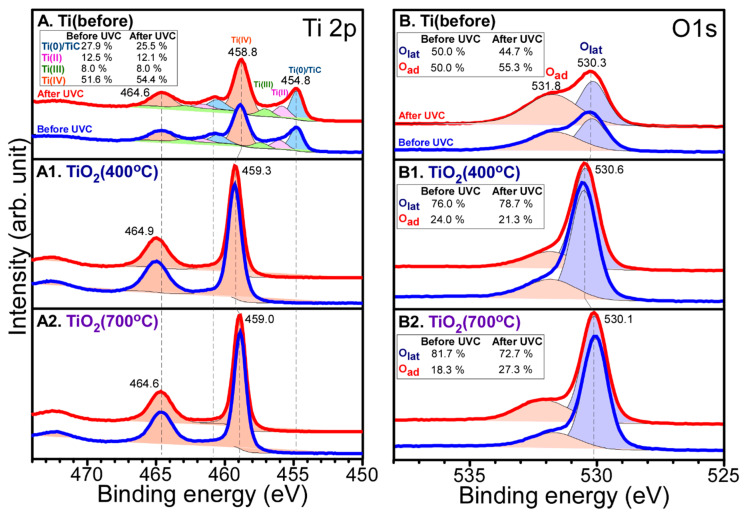
Ti 2p (left column) and O 1s (right column) XPS of: (**A**,**B**) bare Ti; (**A1**,**B1**) Ti (400 °C); (**A2**,**B2**) Ti (700 °C) before (blue) and after (red) CO_2_ reduction. The dot lines are normalized peaks for comparison before and after CO_2_ reduction. Inset tables show relative ratios of the deconvoluted peaks.

**Figure 4:**
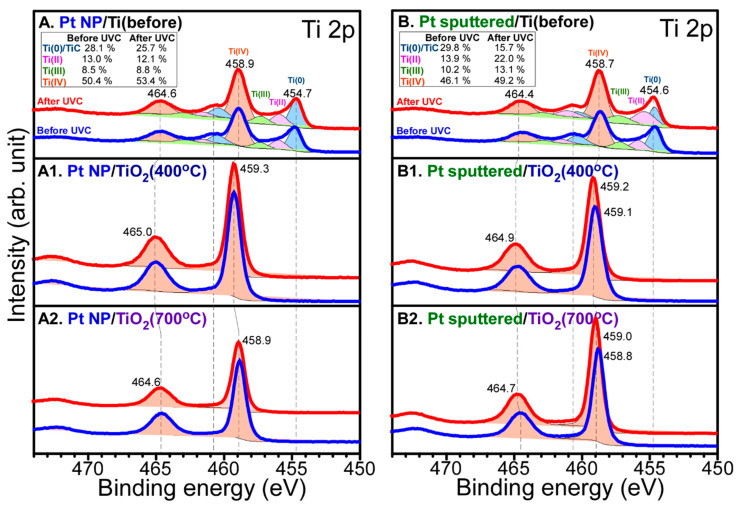
Ti 2p XPS of: (**A**) Pt NP/Ti; (**A1**) Pt NP/Ti (400 °C); (**A2**) Pt NP/Ti (700 °C); (**B**) Pt-sp/Ti; (**B1**) Pt-sp/Ti (400 °C); (**B2**) Pt-sp/Ti (700 °C) samples before (blue) and after (red) CO_2_ reduction. Inset tables show relative ratios of the deconvoluted peaks.

**Figure 5:**
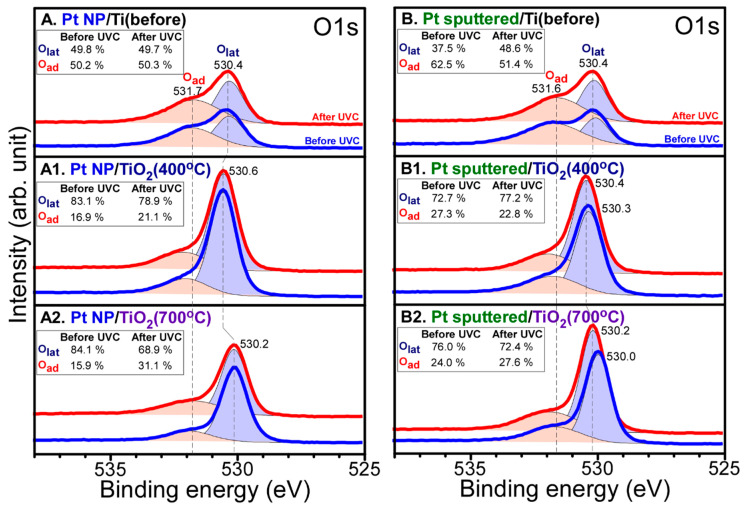
O 1s XPS of: (**A**) Pt NP/Ti; (**A1**) Pt NP/Ti (400 °C); (**A2**) Pt NP/Ti (700 °C); **(B**) Pt-sp/Ti; (**B1**) Pt-sp/Ti (400 °C); (**B2**) Pt-sp/Ti (700 °C) samples before (blue) and after (red) CO_2_ reduction. Inset tables show relative ratios of the deconvoluted peaks.

**Figure 6:**
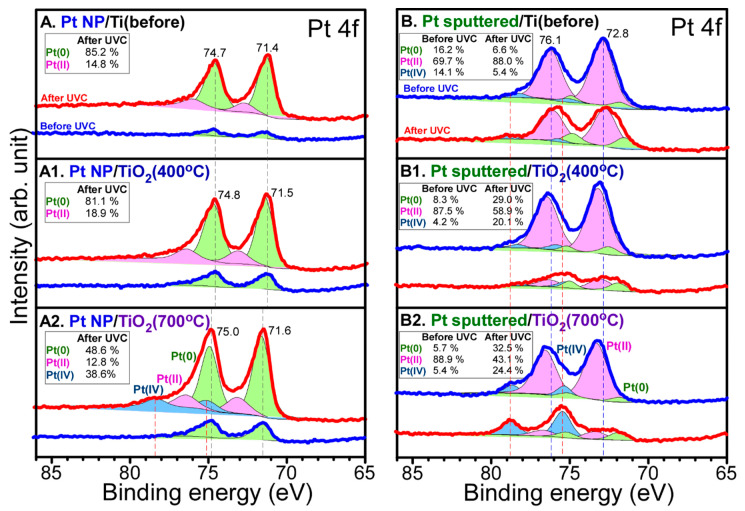
Pt 4f XPS of: (**A**) Pt NP/Ti; (**A1**) Pt NP/Ti (400 °C); (**A2**) Pt NP/Ti (700 °C); (**B**) Pt-sp/Ti; (**B1**) Pt-sp/Ti (400 °C); (**B2**) Pt-sp/Ti (700 °C) samples before (blue) and after (red) CO_2_ reduction. Inset tables show relative ratios of the deconvoluted peaks.

The surface oxidation states were examined by obtaining X-ray photoelectron spectroscopy (XPS) data before and after photocatalytic CO_2_ reduction. All the XPS peaks were deconvoluted for the clear identification of several oxidation states. The relative peak ratios are provided in the inset table in each Figure 3, Figure 4, Figure 5 and Figure 6. Figure 3 shows Ti 2p and O 1s XPS profiles for Pt-free Ti, Ti (400 °C), and Ti (700 °C) samples. The Ti 2p profiles were quite similar before and after CO_2_ reduction. For the Ti 2p XPS for bare Ti in Figure 3A, several peaks were observed at binding energies (BEs) of 454.8, 458.8, and 464.6 eV, and between these peaks. The Ti 2p_1/2_ and Ti 2p_3/2_ peaks were observed at BEs of 464.6 and 458.8 eV, respectively with a spin-orbit splitting of 5.8 eV. This is attributed to Ti(IV) state of TiO_2_ [38,39,40]. Other Ti 2p_1/2_ and Ti 2p_3/2_ peaks were observed around 460.8 and 454.8 eV, respectively. This is attributed to Ti(II) of TiC [38,39,40] whose corresponding C 1s peak was observed at 281.9 eV. In addition, the broadly distributed Ti 2p_3/2_ signals between 454.8 and 459.0 eV could be due to Ti(II,III) oxidation states. For the 400 °C-annealed Ti in Figure 3(A1), substantial change in Ti 2p peak was observed, the Ti 2p_1/2_ and Ti 2p_3/2_ peaks were observed only at BEs of 464.9 and 459.3 eV, respectively. No Ti 2p signals of Ti(II) and Ti(III) species were observed. This indicates that all Ti(II) and Ti(III) species were oxidized to Ti(IV). For the 700 °C-annealed Ti in Figure 3(A2), the Ti 2p_1/2_ and Ti 2p_3/2_ peaks were observed only at BEs of 464.6 and 459.0 eV, respectively. The BEs were shifted by 0.3 eV to a lower BE position, compared with those for the 400 °C-annealed Ti. This implies that the acidity of Ti^4+^ site became lower upon 700 °C annealing.

For the O 1s X-ray photoelectron (XP) spectra of Pt-free Ti, Ti (400 °C) and Ti (700 °C) samples before and after CO_2_ reduction, two broad regions were commonly observed, and the lower and higher BE peaks were attributed to lattice oxygen (O_lat_) and adsorbed surface oxygen species (O_ad_), respectively [29,39,40,41]. The O_ad_/O_lat_ ratio of Ti sample in Figure 3B was found to be much higher than those of Ti (400 °C) and Ti (700 °C) samples, as expected. The O 1s XP BE positions for lattice oxygen were observed to be 530.3, 530.6 and 530.1 eV for Ti, Ti (400 °C) and Ti (700 °C) samples, respectively. However, the O 1s XP BE positions for surface oxygen species were found to be similar for the three different samples. For the O 1s of the Ti (400 °C) sample in Figure 3(B1), the O_ad_/O_lat_ ratio was somewhat decreased after CO_2_ reduction. However, the O_ad_/O_lat_ ratio was increased to be higher for Ti and Ti (700 °C) samples after CO_2_ reduction in Figure 3(B2). This reflects higher CO_2_ reduction activity for Ti and Ti (700 °C) samples, compared with the Ti (400 °C) sample. This is in good consistent with the data shown in Figure 2B.

Figure 4 shows Ti 2p XPS for Pt NP/Ti, Pt NP/Ti (400 °C), Pt NP/Ti (700 °C), Pt-sp/Ti, Pt-sp/Ti (400 °C), and Pt-sp/Ti (700 °C) samples before and after CO_2_ reduction. For the Ti 2p XPS for Pt NP/Ti in Figure 4A, several peaks were observed at binding energies (BEs) of 454.7, 458.9, 464.6 eV, and between these peaks, as observed above. The Ti 2p_1/2_ and Ti 2p_3/2_ peaks were observed at BEs of 464.6 and 458.9 eV, respectively with a spin-orbit splitting of 5.7 eV. This is attributed to Ti(IV) state of TiO_2_ [38,39,40]. Other Ti 2p_1/2_ and Ti 2p_3/2_ peaks were observed around 460.8 and 454.7 eV, respectively. This is attributed to Ti(II) of TiC, as mentioned above [38,39,40]. In addition, the broadly distributed Ti 2p_3/2_ signals between 454.7 and 459.0 eV could be due to Ti(II,III) oxidation states. The Ti 2p XPS showed no critical change after CO_2_ reduction. For the Ti 2p XPS for Pt-sp/Ti in Figure 4B, the Ti 2p XPS profiles were quite similar to those for Pt NP/Ti in Figure 4A. For the Ti 2p XPS for Pt NP/Ti (400 °C) before and after CO_2_ reduction in Figure 4(A1), it was clear that the Ti 2p signals of Ti(0), Ti(II), and Ti(III) completely disappeared. This indicates that all the species were changed to Ti(IV) upon thermal annealing at 400 °C. The Ti 2p_1/2_ and Ti 2p_3/2_ peaks were observed at BEs of 465.0 and 459.3 eV, respectively. The BE was shifted by +0.4 eV to a higher BE position. For the Ti 2p XPS for Pt-sp/(400 °C) in Figure 4(B1), the Ti 2p XPS profiles were also quite similar to those for Pt NP/(400 °C) in Figure 4(A1). For the Ti 2p XPS for Pt NP/Ti (700 °C) before and after CO_2_ reduction in Figure 4(A2), the Ti 2p_1/2_ and Ti 2p_3/2_ peaks were observed at BEs of 464.6 and 458.9 eV, respectively. The BE was shifted by 0.4 eV to a lower BE position, compared with that for the Ti 2p XPS of Pt NP/Ti (400 °C). This indicates that the acidity of Ti^4+^ site became lower upon annealing at a higher temperature. The Ti 2p XPS showed no big change after CO_2_ reduction experiment. For the Ti 2p XPS for Pt-sp/(700 °C) in Figure 4(B2), the Ti 2p XPS profiles were quite similar to those for Pt NP/(700 °C) in Figure 4(A2). On the basis of the Ti 2p XPS results, the natures of Ti sites were quite different for all the samples, meaning that different CO_2_ reduction and HER activities were expected, as discussed above and below.

Figure 5 displays O 1s XP spectra for Pt NP/Ti, Pt NP/Ti (400 °C), Pt NP/Ti (700 °C), Pt-sp/Ti, Pt-sp/Ti (400 °C), and Pt-sp/Ti (700 °C) samples before and after CO_2_ reduction. Two broad regions were commonly observed as discussed above, and the lower and higher BE peaks were attributed to lattice oxygen (O_lat_) and adsorbed surface oxygen species (O_ad_), respectively [29,39,40,41]. The O_ad_/O_lat_ ratios of Pt NP/Ti and Pt-sp/Ti samples were much higher than those of corresponding Ti (400 °C) and Ti (700 °C) samples, due to a thermal treatment effect. The O 1s XP BE positions for lattice oxygen were observed to be 530.4, 530.6, and 530.2 eV for Pt NP/Ti, Pt NP/Ti (400 °C) and Pt NP/Ti (700 °C) samples, respectively. For Pt-sp/Ti, Pt-sp/Ti (400 °C), and Pt-sp/Ti (700 °C) samples, the O 1s XP BE positions were observed to be 530.2, 530.3, and 530.0 eV, respectively. However, the O 1s XP BE positions for surface oxygen species were found to be similar for the three different samples. A major difference in the O 1s profile after CO_2_ reduction was found in the intensity of adsorbed surface oxygen species. The Pt NP/Ti (400 °C) and Pt-sp/Ti (400 °C) samples showed minimal change in the intensity of adsorbed surface oxygen species after CO_2_ reduction, compared with other samples. This reflects that as discussed above, the Ti (400 °C) samples showed the lowest CO_2_ reduction activity, consistent with the data shown in Figure 2C,D.

Figure 6 displays Pt 4f XP spectra for Pt NP/Ti, Pt NP/Ti (400 °C), Pt NP/Ti (700 °C), Pt-sp/Ti, Pt-sp/Ti (400 °C), and Pt-sp/Ti (700 °C) samples before and after CO_2_ reduction. For the Pt NP-loaded samples before CO_2_ reduction, two major peaks were observed at 75.0 and 71.6 eV for Pt NP/Ti (700 °C), attributed to Pt 4f_5/2_ and Pt 4f_7/2_ XPS peaks of metallic Pt, respectively [29,40]. The Pt NP/Ti sample showed at BEs of 74.7 and 71.4 eV for Pt 4f_5/2_ and Pt 4f_7/2_ XPS peaks. The Pt 4f_5/2_ and Pt 4f_7/2_ XPS peaks for the Pt NP/Ti (400 °C) showed BEs of 74.8 and 71.5 eV, respectively. After CO_2_ reduction, the major BE position showed no critical change, but the XPS signals above 75 eV were slightly enhanced. This reflects an increase in Pt^4+^ species showing BEs at 78.8 and 75.3 eV for Pt 4f_5/2_ and Pt 4f_7/2_ XPS peaks. For the sputtered-Pt samples before CO_2_ reduction, a big difference in BE position was found. Two major peaks at 76.6 and 73.3 eV were found for Pt-sp/Ti (700 °C) sample, attributed to Pt 4f_5/2_ and Pt 4f_7/2_ XPS peaks of Pt(II), respectively [29]. In addition, smaller peaks were observed at 78.7 and 75.4 eV, attributed to Pt 4f_5/2_ and Pt 4f_7/2_ XPS peaks of Pt(IV), respectively. Metallic Pt was weakly seen in the spectra [20,41]. This indicates that sputtered Pt were of Pt(II) and Pt(IV) species co-existed as oxidized Pt species. For the Pt-sp/Ti samples before CO_2_ reduction, two major peaks were found at 76.1 and 72.8 eV. For Pt-sp/Ti (400 °C) before CO_2_ reduction, two major peaks were found at 76.4 and 73.1 eV. The Ti support annealed at a higher temperature showed higher BE position for the Pt 4f_5/2_ and Pt 4f_7/2_ XPS peaks of Pt(II). For the Pt-sp/Ti samples after CO_2_ reduction, Pt(II) oxidation species were decreased while Pt(IV) and Pt(0) species were observed to be increased. The change in Pt 4f became more distinct for the Ti support annealed at a higher temperature. For the Pt-sp/Ti (700 °C) sample, Pt(IV) species became dominant and the corresponding Pt 4f_5/2_ and Pt 4f_7/2_ XPS peaks were found at 78.7 and 75.4 eV. For the Ti (700 °C) support, Pt(IV) species were commonly observed to be higher than other Ti supports.

**Figure 7:**
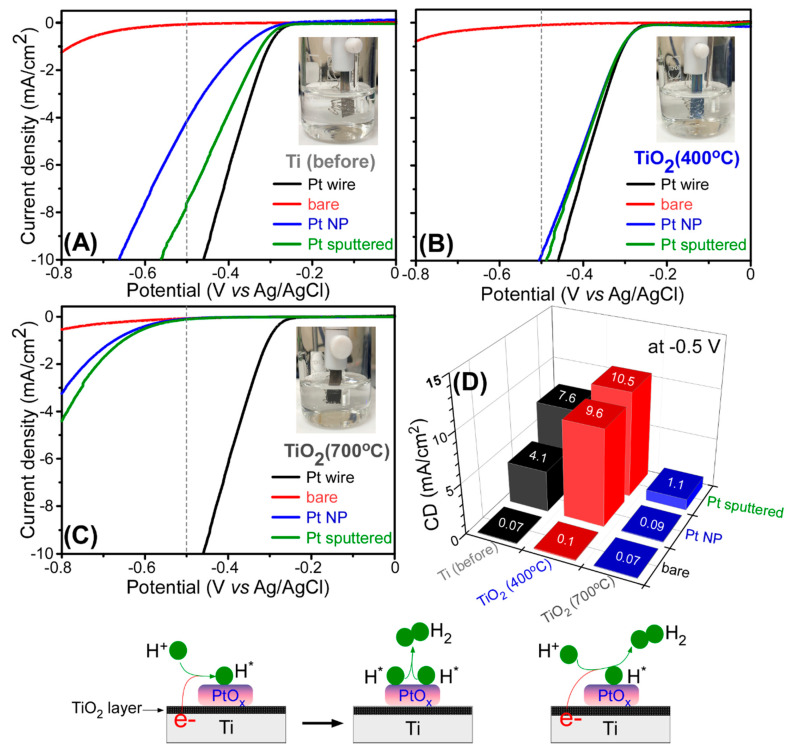
Linear sweep voltammetry (LSV) data at a scan rate of 10 mV/s for: (**A**) bare Ti, Ti (400 °C) and Ti (700 °C); (**B**) Pt NP/Ti, Pt NP/Ti (400 °C) and Pt NP/Ti (700 °C); (**C**) Pt-sp/Ti, Pt-sp/Ti (400 °C) and Pt-sp/Ti (700 °C) samples, and for Pt wire (blank line); (**D**) current density (mA/cm^2^) taken at −0.5 V for the corresponding samples. The HER mechanism is depicted below.

For hydrogen production in a three-electrode electrochemical system, electrochemical linear sweep voltammetry (LSV) was tested to examine electrocatalytic hydrogen evolution reaction (HER) activities for Pt wire, bare Ti, oxidized (400 and 700 °C-annealed) Ti, Pt NP/Ti, Pt NP/Ti (400 °C), Pt NP/Ti (700 °C), Pt-sp/Ti, Pt-sp/Ti (400 °C), and Pt-sp/Ti (700 °C) samples. Pt has popularly been used for hydrogen production and shown good catalytic activity [13,14,15,16,42]. Figure 7 displays the corresponding LSV data (between 0.0 and −0.8 V vs. Ag/AgCl) obtained in 0.1 M H_2_SO_4_ solution. The corresponding current density (CD, mA/cm^2^) taken at −0.5 V was also plotted for direct comparison of the HER activity. It was clearly shown that the CD was found to be considerably dependent on the Pt-loading and thermal treatment temperature of Ti support. The Pt-free bare Ti, Ti (400 °C), and Ti (700 °C) samples showed the poorest activity, showing onset potentials above −0.6 V, compared with Pt-loaded samples. HER CDs were observed to be 0.07, 0.1, and 0.07 mA/cm^2^ at −0.5 V (vs. Ag/AgCl) for Ti, Ti (400 °C), and Ti (700 °C) samples, respectively. For a Pt wire, the onset potential was observed around −0.22 V. For the Pt-loaded Ti and Ti (400 °C) samples (Figure 7A,B, respectively) showed the onset potentials close to that of a Pt wire. However, the Pt-loaded Ti (700 °C) samples (Figure 7C) showed much higher HER onset potential of −0.5 V. This indicates that the HER activity was shown to be much poorer than those of Pt-loaded Ti and Ti (400 °C) samples. The HER activity showed the order of Ti (700 °C) < Ti < Ti (400 °C), whose order was inverse to the CO_2_ reduction. The Ti (400 °C) support showed the highest HER activity and increased upon Pt-loading. This is a clear evidence that catalyst support is very important for improving catalytic activity. For Pt-NP loaded samples, HER CDs were observed to be 4.1, 9.6, and 0.09 mA/cm^2^ at −0.5 V (vs. Ag/AgCl) for Pt-NP/Ti, Pt-NP/Ti (400 °C), and Pt-NP/Ti (700 °C), respectively. For sputtered Pt-loaded samples, HER CDs were observed to be 7.6, 10.5, and 1.1 mA/cm^2^ at −0.5 V (vs. Ag/AgCl), Pt-sp/Ti, Pt-sp/Ti (400 °C), and Pt-sp/Ti (700 °C), respectively. It was common that the sputtered Pt-loaded samples showed higher HER activity compared with the corresponding Pt-NP loaded samples, in good agreement with the literature [33]. Lian et al. also observed same for H_2_ evolution efficiency tests over Pt*^n^*^+^ (*n* = 0, 2, or 3)-Ti^3+^/TiO_2_ and metallic Pt-loaded Ti^3+^/TiO_2_ [33]. XPS data (Figure 6) and HER activity (Figure 7) clearly confirms that the oxidation state of overlayer Pt plays a significant role in improving HER activity. In addition, when the TiO_2_ layer is too thick (in case of the 700 °C-treated samples) the HER activity becomes poor because of poor electrical current flow in an electrochemical reaction.

For the HER mechanism in acidic media, hydrogen is adsorbed on the catalyst surface via H_3_O^+^ + e^−^ → H_ad_ + H_2_O. Then, molecular hydrogen is released from the surface via H_ad_ + H_3_O^+^ + e^−^ → H_2_ + H_2_O or H_ad_ + H_ad_ → H_2_ [43,44,45]. The mechanism is depicted in Figure 7, where the adsorption of H occurs on (or periphery of) PtO_x_ or Pt NPs species. When hydrogen adsorption Gibbs free energy, *ΔG_HX_* is closer to 0 eV the HER activity become higher. When Pt (or PtO_x_) is present, the *ΔG_HX_* appears to be close to 0 and the HER activity becomes enhanced [43,44,45]. The *ΔG_HX_* is determined by Pt oxidation states (or the relative ratio). The highest HER activity was observed for the support with higher Pt(II) species. When Pt(IV) species were higher on the Ti support, the activity was observed to be poorer. The Ti support with Pt(0) species showed somewhat less activity, compared with the support with Pt(II) species. On the basis of the results, the oxidation state of Pt determines *ΔG_HX_* and consequently affects the HER activity. This needs further investigation. In addition to this Pt oxidation state, the thickness of TiO_2_ overlayer is an important factor as depicted in Figure 7. The thick TiO_2_ layer for the 700 °C-treated samples exhibited poor electrical current to result in low HER activity.

## 4. Conclusions

In summary, Pt NPs and sputtered-Pt were loaded on Ti sheets with different oxidation states, and tested for photocatalytic CO_2_ reduction in a closed reaction chamber and electrochemical hydrogen evolution reaction. Two different demonstration tests were performed to precisely examine the roles of Pt and Ti supports in different application areas. XPS studies confirmed that Pt NP were mainly metallic Pt(0) oxidation state, while sputter-deposited Pt were both Pt(II)-O and Pt(IV)-O species. Ti support before thermal annealing showed oxidation states of Ti(II), Ti(III), and Ti(IV) while the thermal (400 and 700 °C)-annealed Ti showed only the Ti(IV) oxidation state. The CO_2_ reduction products were commonly observed to be CO, CH_4_, and CH_3_OH, and the Pt-free Ti (700 °C) sample showed total yields of up to 87.3, 88.0, 26.9 ppm for the formation of CO, CH_4_, and CH_3_OH, respectively under UVC irradiation for 13 h. For bare Pt-free Ti substrates, the activity showed the order of Ti (400 °C) <Ti < bare Ti (700 °C). Pt-loading commonly negated the CO_2_ reduction yields, but CH_4_ selectivity was found to be increased. For electrochemical hydrogen evolution reaction (HER), the Ti (700 °C) support showed the poorest activity and the HER CDs commonly showed the order of Ti (700 °C) < Ti < bare Ti (400 °C), whose order was inverse to the CO_2_ reduction. The Ti (700 °C) support showed poorest HER activity, although Pt was loaded on the support. The Ti (400 °C) support showed the highest HER activity increased upon Pt-loading. HER CDs were observed to be 0.1, 9.6, and 10.5 mA/cm^2^ at −0.5 V (vs. Ag/AgCl) for bare Ti (400 °C), Pt NP/Ti (400 °C) and Pt-sp/Ti (400 °C), respectively. Sputtered Pt-loaded samples showed a higher activity than the corresponding Pt NP-loaded samples. Conclusively, CO_2_ reduction and electrochemical HER activities were mainly determined by the nature of Ti support and Pt oxidation (0,II,IV) species. The present demonstration tests provide valuable information on the design of Pt-overlayer metals and Ti-supports for energy and the environment.

## Figures and Tables

**Table 1:** Photoreactor conditions and reduction yields for reported Pt/Ti-oxide-based catalysts.

Pt/Ti-Oxide Catalysts [Reference]	Photoreactor Conditions	Major Products and Yields
1% Pt-loading on TiO_2_ with {001} facets [27]	300 W Hg-lamp, 0.1 g on 28 cm^2^ watch glass in 350 mL reactor	CH_4_: 2.6 μmol g^−1^ h^−1^ No CO
Pt (0.2 wt.%)/TiO_2_: impregnation and thermal treatment [34]	0.1 g catalyst, 423 K, 400 W Hg lamp, CO_2_ flow (4.5 mL/min) saturated with H_2_O vapor	CH_4_: 1.46 μmol g^−1^ h^−1^ H_2_: 5.28 μmol g^−1^ h^−1^
Pt (1.82 nm)/TiO_2_ NPs [24]	132 mL stainless steel reactor, 500 W Xe lamp, 20 mg catalyst, 5 mL H_2_O	CH_4_: 60.1 μmol g^−1^ h^−1^ C_2_H_6_: 2.8 μmol g^−1^ h^−1^ H_2_: 87.5 μmol g^−1^ h^−1^
1.5 wt.% Pt/TiO_2_ photocatalyst [35]	UV 8 W Hg lamp (peak intensity at 254 nm) 0.1 g catalyst, 10 mL H_2_O, Pressured CO_2_ in the 348 mL reactor	CO: 7 μmol g^−1^ for 12 h CH_4_: 15 μmol g^−1^ for 12 h H_2_: 270 μmol g^−1^ for 12 h
Pt (3–4 nm)/TiO_2_ nanosheet porous film [36]	300 W Hg lamp (10.4 mW/cm^2^) 20 mL 0.1 mol/L KHCO_3_ solution	CH_4_: 20.5 ppm cm^−1^ h^−1^
Pt on ultrathin TiO_2_ [20]	10 mg catalyst, 50 cm^3^ of chamber volume. 300 W Xe lamp	CH_4_: 47 μmol for 10 h CO: 35 μmol for 10 h
Pt-loaded TiO_2_ spheres (>500 μm) [19]	200 mg catalyst, 100 μL of DI water, pressurized CO_2_ (50 PSI), UV (20 mW/cm^2^, 254 nm)	CO: 18 μmol g^−1^ h^−1^ CH_4_: 3.5 μmol g^−1^ h^−1^ H_2_: 230 μmol g^−1^ h^−1^
Pt NPs (5–12 nm) on TiO_2_ nanofibers through electrospinning [10]	5 mg catalyst on 2 cm × 2 cm glass, 500 W Xe lamp, 0.1 mL of deionized water, 90 mL gastight reactor	CO: 0.08 μmol h^−1^ CH_4_: 0.42 μmol g^−1^ h^−1^
Pt^2+^–Pt^0^/TiO_2_ NPs by sol-gel method [37]	300 W Xe arc lamp, 0.1 g on glass-fiber cloth in 85 mL reactor, a mixture of CO_2_, and water vapor flow	CO: 20 μmol g^−1^ for 14 h CH_4_: 264 μmol g^−1^ for 7 h H_2_: 2763 μmol g^−1^ for 7 h
Sputter deposited Pt (1 nm) on TiO_2_ by aerosol chemical vapor deposition [22]	400 W Xe lamp (250–388 nm, 19.6 mW/cm^2^), a mixture of CO_2_ and water vapor, flow reactor system	CH_4_: 1361 μmol g^−1^ h^−1^ CO: 200 μmol g^−1^ h^−1^
Platinum-impregnated P25, Pt/TiO_2_ [17]	UV-curing 100 W high-pressure Hg lamp (170 mW cm^−2^), 353 and 423 K, gas-phase continuous flow reactor.	CH_4_: 1.08 μmol g^−1^ h^−1^ H_2_: 11.4 μmol g^−1^ h^−1^
Pt/TiO_2_ nanotube [21]	a 300 W high-pressure Hg lamp (wavelength 365 nm), a fixed-bed photocatalysis reactor, 50 mg on the flat quartz plate	CH_4_ yield with 0.0565 μmol h^−1^ g^−1^ after 7 h UV irradiation.
